# Methods for diagnosing bile acid malabsorption: a systematic review

**DOI:** 10.1186/s12876-019-1102-1

**Published:** 2019-11-14

**Authors:** Ivan Lyutakov, Francesco Ursini, Plamen Penchev, Giacomo Caio, Antonio Carroccio, Umberto Volta, Roberto De Giorgio

**Affiliations:** 1 Clinic of Gastroenterology, University Hospital “Tsaritsa Yoanna – ISUL”, Sofia, Bulgaria; 2 Department of Medical Sciences, University of Ferrara, Ferrara, Italy; 3 DiBiMIS University of Palermo, Palermo, Italy; 4 Internal Medicine, Giovanni Paolo II Hospital, Sciacca (ASP Agrigento), Sciacca, Italy; 5 Department of Medical and Surgical Sciences, University of Bologna, Bologna, Italy

**Keywords:** Bile acid malabsorption, Biomarkers, Chronic diarrhea, Diagnostic accuracy

## Abstract

**Background:**

Bile acid malabsorption (BAM) and bile acid-related diarrhea represent an under-recognized cause of chronic diarrhea mainly because of limited guidance on appropriate diagnostic and laboratory tests. We aimed to perform a systematic review of the literature in order to identify and compare the diagnostic accuracy of different diagnostic methods for patients with BAM, despite a proven gold standard test is still lacking.

**Methods:**

A PubMed literature review and a manual search were carried out. Relevant full papers, evaluating the diagnostic accuracy of different methods for BAM, were assessed. Available data were analyzed to estimate the sensitivity and specificity of each published test.

**Results:**

Overall, more than one test was considered in published papers on BAM. The search strategy retrieved 574 articles; of these, only 16 were full papers (with a total of 2.332 patients) included in the final review. Specifically, *n* = 8 studies used ^75^Selenium-homotaurocholic-acid-test (^75^SeHCAT) with a < 10% retention threshold; n = 8 studies evaluated fasting serum 7-α-hydroxy-4-cholesten-3-one (C4); *n* = 3 studies involved total fecal bile acid (BA) excretion over 48 h; *n* = 4 studies assessed fibroblast growth factor 19 (FGF19). ^75^SeHCAT showed an average sensitivity and specificity of 87.32 and 93.2%, respectively, followed by serum C4 (85.2 and 71.1%) and total fecal BA (66.6 and 79.3%). Fasting serum FGF19 had the lowest sensitivity and specificity (63.8 and 72.3%). All the extracted data were associated with substantial heterogeneity.

**Conclusions:**

Our systematic review indicates that ^75^SeHCAT has the highest diagnostic accuracy for BAM, followed by serum C4 assay. The diagnostic yield of fecal BA and FGF19 assays is still under investigation. Our review reinforces the need for novel biomarkers aimed to an objective detection of BAM and therefore improving the management of this condition.

## Background

A chronic, watery diarrhea is a common occurrence in patients with bile acid malabsorption (BAM), a condition known to be characterized by significant clinical heterogeneity [1]. BAM is known to worsen the patients’ quality of life and be a challenge for many healthcare services because of direct and indirect costs [2, 3]. Recently, several research groups have focused their interest to altered bile acid (BA) excretion and reabsorption as a prominent cause of chronic diarrhea [2]. Nonetheless, the mechanisms leading to excessive accumulation of BAs are still only partly defined. From a physiological standpoint, BAs are small amphipathic molecules synthesized by the liver and secreted with meals in order to absorb dietary fats and fat-soluble vitamins [4]. Approximately 95% of the unbound BAs are reabsorbed through the ileal BA transporters and travel through the portal circulation to the liver for recycling [3, 4]. After reabsorption, BAs stimulate the nuclear farsenoid X receptor (FXR), which acts as main nuclear BA receptor and increases fibroblast growth factor 19 (FGF19) [3]. FGF19 is secreted from ileal enterocytes, acts as an entero endocrine hormone to decrease hepatic BA synthesis by downregulating the limiting rate enzyme, 7 α-hydroxy-4-cholesten-3-one (C4) [5]. C4 is an important metabolic intermediate which controls cholesterol 7-hydroxylase (CYP7A1), the enzyme involved in BA synthesis starting from cholesterol [5]. BAM occurs when excessive BAs are present in the colon as a result of an imbalance of the entero-hepatic circulation [4].

In the clinical setting, current main methods to diagnose BAM include the scintigraphic method ^75^Selenium-homotaurocholic acid test (^75^SeHCAT), the serum levels of 7-α-hydroxy-4-cholesten-3-one (C4) and FGF19, whereas bile acid (BA) excretion can be determined in stools over 48 h [2, 3]. Studies using SeHCAT have identified four subtypes of BAM generally recognized as: a) ileal disease preventing BA reabsorption, e.g. Crohn’s disease, ileal resection, and radiation ileitis (type 1); b) “primary” BAM associated with increased BA production, likely playing a role in a subset of patients with functional diarrhea and/or diarrhea-predominant irritable bowel syndrome (IBS-D) (type 2); c) normal terminal ileum BA re-absorption in the context of various conditions, e.g. chronic pancreatitis, celiac disease, post-cholecystectomy and microscopic colitis (type 3) [3]; and, finally, d) excessive hepatic BA synthesis in patients taking metformin or with hypertriglyceridemia, without any clear source of impaired BA re-absorption (type 4) [6]. However, ^75^SeHCAT has many drawbacks, such as limited availability (can be performed in some tertiary referral centers), needs a nuclear medicine unit where the patient has to be investigated in two occasions (on day 0 and after 7 days), has an unavoidable exposure of radiation (equivalent to 370 KBq or 280 keV) and it is also expensive. Because of these reasons, other diagnostic methods have gained attention in the clinical setting. Fasting serum C4 is a direct measure of hepatic BA synthesis, while FGF19 represents an indirect measure of ileal bile acid reabsorption and provides feedback inhibition on hepatic BA synthesis [7, 8]. Therefore, when BAs are reabsorbed, more FGF19 is released from the enterocyte and serum C4 is decreased, reflecting a decreased hepatic BA synthesis [3, 5]. Several studies have been conducted with these new biomarkers documenting the occurrence of BAM in patients with chronic diarrhea [9–11].

Physicians are challenged by the lack of reference methods to establish a correct diagnosis of BAM and treat (e.g. with cholestyramine, colesevelam or colestipol) accordingly patients with chronic diarrhea related to this condition [12]. Thus, the present systematic review was intended to evaluate and compare the available evidence on the diagnostic accuracy (i.e. sensitivity and specificity) of different methods, in order to help physicians choosing the best suitable tests to objective identification of BAM in patients with chronic diarrhea.

## Methods

### Search strategy

A systematic review of the literature was performed in order to evaluate the available data on the diagnostic accuracy on the ^75^SeHCAT and compare it with other non-invasive techniques including FGF19, C4 and total fecal BA excretion for diagnosing BAM in patients with chronic diarrhea. For this manuscript preparation we followed the “Diagnostic test accuracy systematic reviews from Joanna Briggs Institute Reviewer’s Manual” [13]. MedLine (via PubMed) databases were searched up to June 2019.

The main search in MedLine was conducted using the search string (“Diarrh*” OR “Irritable bowel syndrome” OR “Inflammatory bowel disease” OR “IBS*” OR “IBD” OR “Microscopic colitis” OR “MC” OR “Lymphocytic colitis” OR “Collagenous colitis” OR “LC” OR “CC” OR “Ulcerative colitis “OR “Crohn’s disease “OR “CD “OR “UC “OR “Celiac disease “OR “Coeliac disease “OR “Bile acid diarrhea “OR “Terminal ileitis “OR “FBD “OR “functional dyspepsia” OR “Bile Acid Synthesis” OR “Functional bowel disorder “OR “ileal resection” OR “Bile acid pool” OR “Bile acid malabsorption “OR “BAM “OR “IBAM” OR “PBAM” OR “malabsorb*” OR “Bile Acids and Salts” OR “bile acid-gut microbiome” OR “Primary bile acid diarrhoea “OR “Functional GI” OR “Bile salt malabsorption“) AND (“SeHCAT” OR “Se-HCAT” OR “75SeHCAT” OR “Se-75″ OR “75-SeHCAT” OR “SE75“ OR “75-selenium homotaurocholic acid “OR “selenium-labelled homotaurocholic acid test” OR “75selenium homotaurocholic acid test “OR “75-selenium homotaurocholic acid test “OR “bile salt receptor” OR “total faecal bile acid “OR “TFBA “OR “Fecal bile acids “OR “total faecal BA in 48h” OR “48h. total faecal bile acid “OR “radiolabelled bile salt retention” OR “23-seleno-25-homotaurocholic acid “OR “farnesoid X receptor “OR “FXR “OR “biomarkers for BAM” OR “fibroblast growth factor 19 “OR “fibroblast growth factor-19 “OR “FGF19 “OR “Serum FGF19” OR “FGF-19 “OR “Total BA in feces “OR “Bile acids in feces “OR “Bile Salt Hydrolase” OR “CYP7A1” OR “nuclear pregnane X receptor” OR “Se-HCAT “OR “14C-glycocholate” OR “Se-75 “OR “selenium homocholic acid taurine “OR “pregnane X receptor” OR “TGR5” OR “Fecal total and individual BAs” OR “7a-hydroxy-4-cholesten-3-one “OR “C4 “OR “Total faecal bile acid “OR “Glycocholate” OR “bile acid precursor” OR “7αOH-4-cholesten-3-one “OR “LC-MC” OR “Primary bile acids in a single fecal” OR “7-α-Hydroxy-4-Cholesten-3-One “OR “7 alfa Hydroxy 4 cholesten 3 one” OR “7-alfa*” OR “7-α*”) AND (“Sensitivity “OR “Specificity “OR “Accuracy “OR “Diagnostic accuracy “OR “Diagnostic yield “OR “PPV “OR “NPV “OR “Positive predictive value “OR “Negative predictive value “OR “Overall accuracy “OR “cost-effectiveness “OR “clinical effectiveness “OR “Clinical assessment “OR “Reference standard “OR “True-negative “OR “True-positive “OR “diagnostic usefulness”).

In addition, applicable keywords were used in different combinations for manual search and bibliography of the selected articles in order to improve the sensitivity of the search strategy. The search was designed and performed by two authors (IL, FU).

### Inclusion criteria and study selection

We included in the final review, studies (full-text articles) meeting the following inclusion criteria:

1. Study design: randomized controlled trials (RCT), observational cross-sectional, case-control, prospective and retrospective studies;
2. Population: studies involving human subjects above age of 18 with chronic diarrhea;
3. Intervention: Comparing the diagnostic sensitivity and specificity of different diagnostic tests for BAM including ^75^SeHCAT, fasting serum C4, fasting serum FGF19 and total fecal BAs;
4. Outcome: Evaluation of the diagnostic yield of different methods for BAM.

### Exclusion criteria

For this study we applied the following exclusion criteria: (a) patients who presented incomplete data; (b) history of previous surgery (i. e., vagotomy, gastrectomy or bariatric surgery for obesity); (c) other types of enteropathies (including parasitic or acute diarrhea caused from infection); (d) drop out during follow-up; (e) studies that included subjects < 18 years of age or those conducted in patients with history of cholecystectomy; (f) radiation enteritis; (g) diverticulitis; (h) *Clostridium difficile* infection; (i) infectious colitis; (j) ischemic colitis; (k) neoplastic diseases including neuroendocrine tumors; (l) laxative abuse; (m) small intestine bacterial overgrowth (SIBO); (n) gut dysmotility; (o) immune deficiency syndrome; (p) carbohydrate malabsorption; (q) hyperthyroidism; (r) pancreatic disorders including chronic pancreatitis, pancreatic cancers and exocrine pancreas deficiency; (s) studies published in language different from English; (t) abstracts / posters or letters in journals; and, finally, (u) animal studies.

Two authors (IL and FU) screened independently titles and abstracts of retrieved records for inclusion in the systematic review. A three-stage search strategy was performed, including an initial search of the selected database using the pre-specified search string to identify additional relevant keywords and index terms. The second thorough search across all included articles was performed as full text evaluation. A final review of the reference list was conducted to identify any missed studies.

**Fig. 1:**
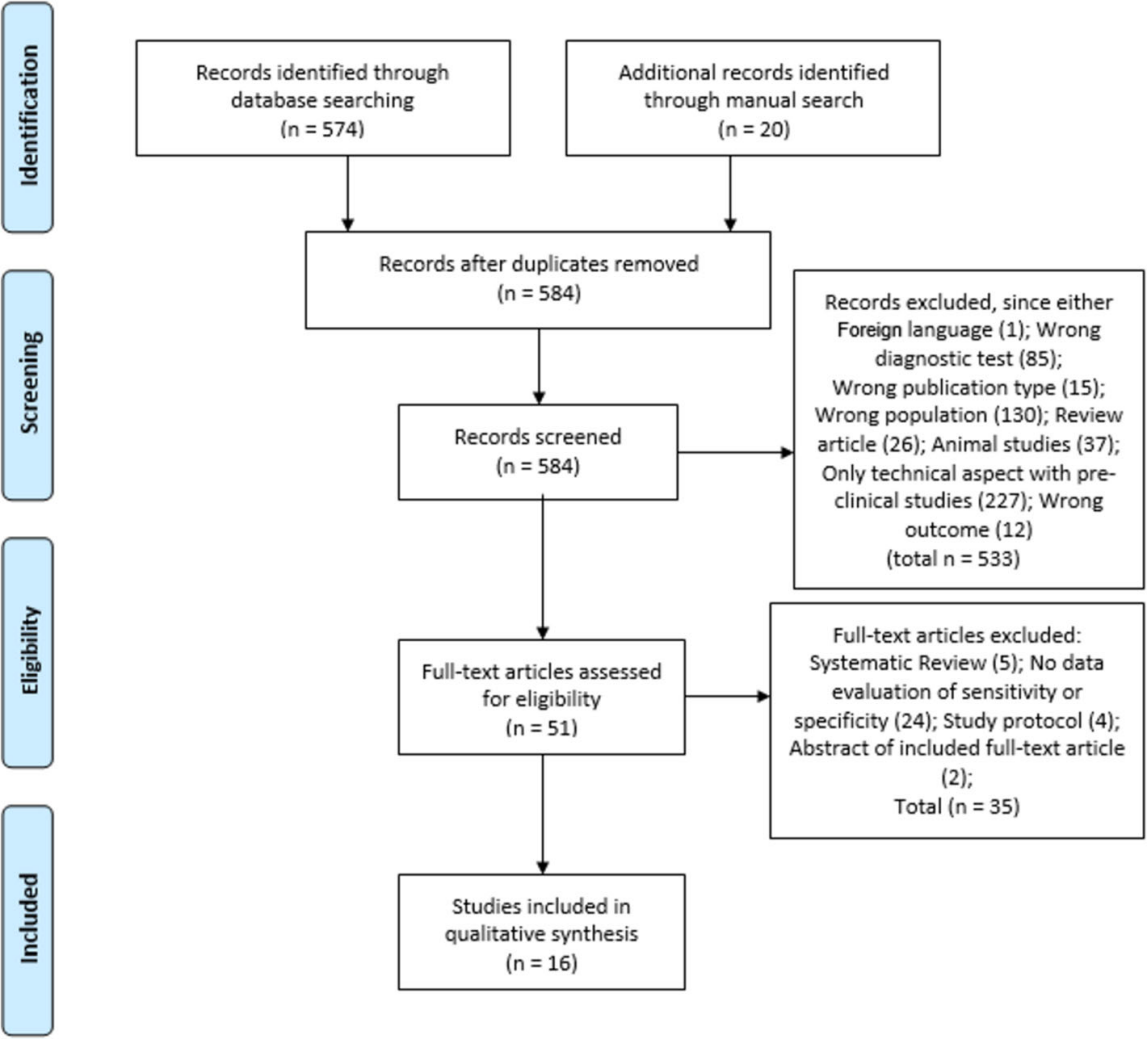
The selection process has been based on key steps including identification, screening, eligibility and, finally, studies actually included in this systematic review according to the illustrated flowchart

After the screening phase, the same two reviewers independently evaluated the remaining articles to determine eligibility according to the inclusion and exclusion criteria. Disagreements among the two reviewers were resolved by discussion with a third senior reviewer (RDG) until reaching a final consensus. Quality was independently appraised by the authors (RDG, UV, GC, AC and PP). A full flowchart of the study selection process is illustrated in Fig. 1.

## Results

### Search results and study description

The primary search strategy initially retrieved 574 records. Twenty additional articles were added by manual search of relevant references and check of bibliography. Ten records that were duplicated have been removed. After screening titles and abstracts, a total of 533 studies were excluded because of inappropriate / low sensitive diagnostic tests (e.g., ^14^C glycocholate breath test), non-English language, review articles, preclinical studies or clinical studies dealing with inadequate patient selection (e.g., those without chronic diarrhea) or wrong outcome (i.e., patients evaluated in terms of treatment response, not for test accuracy). Amongst the remaining 51 studies selected for final examination, only 16 articles were included in this systematic review because they investigated the diagnostic accuracy (i.e. sensitivity and specificity) of the diagnostic methods to objectively detect BAM. Causes of exclusion at each step were summarized in Fig. 1. The main findings of selected articles have been outlined in Table 1.

**Table 1:** Characteristics and main findings of included articles. Se - sensitivity, Sp – Specificity

Author, (year), reference	Country	Study design	Number of patients /females	Study population	Method used to identify BAM	Se	Sp	Main findings
Vijayvargiya. P, 2019 [14]	USA	Retrospective case-control study	*n* = 124(109)	IBS-D, IBS-C and healthy volunteers	Total fecal 48-h BA in combination with primary fecal BAs	49%	91%	Primary BAs > 10% identified patients with increased fecal weight (sensitivity 49% and specificity 91%) and rapid colonic transit (sensitivity 48% and specificity 87%
Vijayvargiya. P, 2019 [15]	USA	Retrospective case-control study	*n* = 220(171)	HV, IBS-D and IBS-C	Fecal bile acids and fecal fat	76%	72%	Reduced total and primary fecal bile acids and increased fecal lithocholic acid were significant predictors of decreased fecal weight, frequency and consistency.
Battat R., 2019 [16]	USA	Prospectively cross-sectional study	*n* = 78 (47)	Crohn’s disease (CD) - IR, NR-CD and UC	C4	90%	84%	A cutoff concentration of C4 of 48.3 ng/mL or greater identified patients with diarrhea attributable to BAM with 90.9% sensitivity, 84.4% specificity
Donato L., 2018 [17]	USA	Prospectively cross-sectional study	*n* = 184 (110)	IBS-C, IBS-D, Healthy subjects	C4	82%	53%	Higher levels of C4 was found in patients with BAM compared to those without BAM with sensitivity/specificity of 82%/53%.
Vijayvargiya P., 2017 [18]	USA	Prospectively cross-sectional study	*n* = 101 (*n* = 83)	IBS-D	C4 and FGF19	50%	65%	Data demonstrated a higher specificity (83%) with a higher cut-off of 52.5 ng/mL.
Camilleri M., 2014 [19]	USA	Prospectively cross-sectional study	*n* = 124 (111)	IBS-D, IBS-C and HS	Total fecal 48-h BA in combination with primary fecal Bas	75%	75%	Estimated the specificity of the individual traits or models at 60% sensitivity for discriminating between the groups, with specificity ranging from 75% for IBS-D versus health, to 90% for IBS-D versus IBS-C
Pattni S., 2013 [11]	UK	Prospectively cross-sectional study	*n* = 72 (47)	Chronic diarrhoea of unknown aetiology	FGF19 compare to SeHCAT	67%	77%	NPV and PPV of FGF19 ≤ 145 pg/mL for a SeHCAT < 10% were 82 and 61%. Data suggest that FGF19 could predict response to sequestrant therapy
Pattni S., 2012 [20]	UK	Prospectively cross-sectional study	*n* = 258 (180)	patients with chronic diarrhea	FGF-19 + C4	58% 74%	79% 72%	The sensitivity and specificity of FGF19 at 145 pg/ml for detecting a C4 level > 28 ng/ml were 58 and 79%, respectively. For C4 > 60 ng/ml, these were 74 and 72%;
Brydon WG., 2011 [21]	Scotland	Prospectively cross-sectional study	*n* = 196 (108)	Patients with unexplained diarrhea	C4 BAM type 1 C4 BAM type 2 compare to SeHCAT	97% 90%	74% 77%	ROC analysis yielded a sensitivity/specificity of 90%/77% for type 1 BAM (ileal disease/resection) and 97%/74% for type 2 BAM (idiopathic) using 30 ng/mL as the upper limit of normal for serum C4
Lenicek M., 2011 [22]	Czech Republic	Prospectively cross-sectional study	*n* = 466 (267)	Crohn’s disease, Ilear Crohn’s resection and Healthy Subjects	FGF19 and C4	80%	68%	FGF19 levels maximizing precision was set to < 60 ng/L. In this case, the sensitivity and specificity of FGF19 as a marker of BAM reached 80 and 68%. BAM was also present in a substantial number of the patients with CD
Sauter GH, 1999 [23]	Germany	Prospective cross-sectional study	*n* = 129 (68)	HS + chronic diarrhea of unknown origin	C4 Compare to SeHCAT	90%	79%	75SeHCAT test yielded the same results in 19/23 (83%) patients. BAM was identified by an increase of C4 in serum with a sensitivity of 90% and a specificity of 79%.
Brydon WG, 1996 [24]	UK	Prospective case-control study	*n* = 164 (108)	chronic diarrhoea investigated prospectively	C4 Compare to SeHCAT	NPV 74% 100%	PPV 94% 96%	The positive predictive value of serum C4 was 74%. The high negative predictive value (98%) of serum c4
Balzer K, 1993 [25]	Germany	Prospective case-control study	*n* = 64	HS and patients with ileal disease or resection	SeHCAT	80%	96%	75SeHCAT retention test: sensitivity 80%, specificity 98%, accuracy 89%
Scheurlen C, 1988 [26]	Germany	Prospective case-control study	*n* = 64	Crohn’s disease	SeHCAT	59.6%	100%	At a specificity of 100% the sensitivity of the SeHCAT test was 59.6% and the efficiency was 67.2%.
Sciarretta G, 1987, [27]	Italy	Prospective case-control study	*n* = 46 (26)	healthy volunteers, distal ileum resection, Crohn’s and chronic diarrhea	SeHCAT	100%	94%	SeHCAT found to be the most suitable for differentiating between the groups, giving the 75SeHCAT test a 94% sensitivity and a 100% specificity. The data show that this test is a valid indicator of bile acid loss.
Merrick MV, 1985 [28]	UK	Prospective case-control study	*n* = 42	IBS and Ileal resection	SeHCAT	97%	80–99%	The diagnosis was established by measuring the proportion of SeHCAT, a synthetic bile salt, retained 1 week after oral administration of a tracer dose of less than 100F/kg of the compound labelled with 40 kBq (1. Ci) of selenium-75.

### Risk of bias assessment

The remaining 51 studies were critically appraised using the “The Joanna Briggs Institute Critical Appraisal tools” for systematic reviews. A checklist for diagnostic test accuracy studies and results were summarized in Table 2 [29]. About 87.5% of the studies had a prospective design, whereas only 12.5% had a retrospective design. Approximately 50% of the studies reported the diagnosis of BAM based on a 7-day ^75^SeHCAT retention test. The other 50% of the studies has identified BAM base on C4, FGF19 and fecal BA assay. Only 25% of the included studies compared head-to-head ^75^SeHCAT vs. other tests (“biomarkers”) for their diagnostic accuracy in the same study design and population. The overall risk of bias (i.e., downsizing diagnostic accuracy) was low to moderate in all studies.

**Table 2:** JBI Critical Appraisal Checklist for Diagnostic Test Accuracy Studies

Author, (year), reference	1. Was a consecutive or random sample of patients enrolled?	2. Was a case control design avoided?	3. Did the study avoid inappropriate exclusions?	4. Were the index test results interpreted without knowledge of the results of the reference standard?	5. If a threshold was used, was it pre-specified?	6. Is the reference standard likely to correctly classify the target condition?	7. Were the reference standard results interpreted without knowledge of the results of the index test?	8. Was there an appropriate interval between index test and reference standard?	9. Did all patients receive the same reference standard?	10. Were all patients included in the analysis?	Quality rating
Vijayvargiya. P, 2019 [14]	No	No	Yes	Yes	Yes	Yes	Yes	Yes	Yes	Yes	Include
Vijayvargiya. P, 2019 [15]	No	No	Yes	Yes	Yes	No	Yes	Yes	Yes	Yes	Include
Battat R., 2019 [16]	Yes	Yes	Yes	Yes	Yes	No	No	Yes	Yes	Yes	Include
Donato L., 2018 [17]	Yes	Yes	Yes	No	Yes	Yes	No	Yes	No	Yes	Include
Vijayvargiya P., 2017 [18]	Yes	Yes	Yes	No	Yes	Yes	No	Yes	No	Yes	Include
Camilleri M., 2014 [19]	Yes	Yes	Yes	Yes	Yes	No	No	Yes	Yes	Yes	Include
Pattni S., 2013 [11]	Yes	Yes	Yes	Yes	Yes	Yes	Yes	Yes	Yes	Yes	Include
Pattni S., 2012 [20]	Yes	Yes	Yes	No	Yes	No	No	Yes	Yes	Yes	Include
Brydon WG., 2011 [21]	Yes	Yes	Yes	No	Yes	No	No	Yes	Yes	Yes	Include
Lenicek M., 2011 [22]	Yes	Yes	Yes	Yes	Yes	No	No	Yes	Yes	Yes	Include
Sauter GH, 1999 [23]	No	Yes	Yes	No	Yes	Yes	Yes	No	Yes	Yes	Include
Brydon WG, 1996 [24]	No	No	Yes	Yes	Yes	Yes	Yes	Yes	Yes	Yes	Include
Balzer K, 1993 [25]	Yes	No	Yes	Yes	No	Yes	Yes	Yes	Yes	Yes	Include
Scheurlen C, 1988 [26]	No	No	Yes	Yes	Yes	Yes	Yes	Yes	Yes	Yes	Include
Sciarretta G, 1987, [27]	Yes	No	Yes	Yes	No	YEs	Yes	Yes	Yes	Yes	Include
Merrick MV, 1985 [28]	No	Yes	Yes	Yes	No	Yes	Yes	Yes	Yes	Yes	Include

### Systematic review of the overall diagnostic accuracy

The total number of patients from the included studies were *n* = 2332, with *n* = 1520 (65%) females. The results from the systematic review are shown in (Table 1). Studies were divided into groups according to the diagnostic test that they used to measure BAM. A total of 8 studies measured ^75^SeHCAT retention with a < 10% of cut-off value; 8 studies assessed serum C4; 4 studies FGF19 and 3 studies determined total fecal BA in 48 h. It should be noted that 8 studies included more than one measurements of interest for this systematic review and 4 (25%) studies compared directly the diagnostic accuracy between ^75^SeHCAT and the other techniques (C4, FGF19 or total fecal bile acids). For each test, the diagnostic accuracy was defined and directly extracted from every article included in the analysis as the sensitivity and specificity that the indexed test has to confirmed diagnosis of BAM in patients with chronic diarrhea associated to main diagnoses such as functional diarrhea, IBS-D, Crohn’s disease, ulcerative colitis and ileal resection. This diagnostic accuracy can be interpreted as the probability of establishing BAM in the evaluated population.

From all the studies, the highest diagnostic yield was observed with ^75^SeHCAT showing an average reported sensitivity and specificity of 87.32 and 93.2%, followed by serum C4 with 85.2 and 71.1%, respectively. The diagnostic accuracy of total fecal BA in 48 h reached an average sensitivity and specificity of 66.6 and 79.3%, respectively. Fasting serum FGF19 demonstrated the lowest diagnostic yield (63.75 and 72.25% of sensitivity and specificity). Except from C4, however, the heterogeneity was high for all diagnostic tests indicating a high degree of variability across the majority of studies that cannot be explained by chance.

### Diagnostic accuracy of ^75^SeHCAT

^75^SeHCAT is a technique aimed to identify the amount of labeled radioactive Selenium (Se) retained in the body as a result of BA reabsorption. The more conjugated Se is retained, the least BA is lost in the intestinal lumen (i.e., colon). Thus, Se retention of < 10% can be considered an index of BAM-related chronic diarrhea. Based on the results of ^75^SeHCAT measurements BAM can be categorized into different degrees of severity: 0–5% (severe); 5–10% (moderate); and 10–15% (mild). Using ^75^SeHCAT with 10% retention cut-off, the range of sensitivity and specificity were 59.6 to 100% (mean of 87.32%) and 80 to 100% (mean of 93.2%), respectively, among all relevant studies. In the milestone paper by Sciarretta et al. reported that ^75^SeHCAT test had a high sensitivity and specificity (94 and 100%, respectively) in discriminating different subsets of patients with chronic diarrhea vs. healthy subjects [27]. Scheurlen et al. used ^75^SeHCAT to establish the actual occurrence of BAM in patients with Crohn’s disease. The authors investigated a cohort of 64 patients with Crohn’s ileitis and showed in about 31 of them (48.4%) had a true positive tests, whereas 33 patients (51.6%) had a negative test. However, in 21 of the latter 33 patients (63.6%) the test yielded a false negative result, since ileal inflammation or resection was confirmed by radiology or endoscopy. In 14 of those 21 patients with false negative test a stenosis of the intestinal lumen was diagnosed. In this study, therefore, ^75^SeHCAT sensitivity and specificity were the lowest reported in the literature being 59.6 and 67.2%, respectively [30].

### Subgroup analysis for diagnostic accuracy of the C4

C4 is measured by high performance liquid chromatography in the serum of patients with suspected BAM. In most studies, C4 concentration > 48.3 ng/mL is considered a positive cut-off to identify patients with diarrhea attributable to BAM. This cut-off value has been applied in eight studies with a test sensitivity and specificity ranging from 74 to 97% (mean 85.2%) and 53–94% (mean 71.1%), respectively. Compared to < 10% ^75^SeHCAT retention, Vijayvargiya et al. confirmed that serum C4 assay demonstrated 90, 79, 73, and 92% sensitivity, specificity, positive predictive value (PPV) and negative predictive value (NPV), respectively [18]. Also, higher levels of C4 were found in patients with BAM compared to those without BAM with sensitivity/specificity of 82 and 53% [17].

### Subgroup analysis for diagnostic accuracy of the 48 h total fecal Bas

Another test used to identify BAM is represented by the BA assessment (via high performance liquid chromatography) in the feces of patients with chronic diarrhea. As in previous tests, a cut-off of primary BAs > 10% correlates with increased fecal weight and rapid colonic transit [14]. Three studies based on 468 patients with IBS-D and -C showed a sensitivity and specificity of fecal BAs ranging from 49 to 76% (mean 66.6%) and 75–91% (mean 79.3%). Vijayvargiya et al. found that total fecal 48-h BA alone, or in combination with percentage of primary fecal BAs, identified patients with increased fecal weight with an AUROC of 0.86. In contrast, primary fecal BA alone identified patients with increased fecal weight with an AUROC of 0.73, whereas total fecal 48-h BA alone identified patients with increased colonic transit with an AUROC of 0.65 and percentage of primary fecal BA alone identified patients with increased colonic transit with an AUROC of 0.69 [14].

### Subgroup analysis for diagnostic accuracy of the FGF19

Finally, another method to unravel BAM is FGF19 which is measured by enzyme-linked immunosorbent assay (ELISA) in the serum of patients with chronic diarrhea. Notably, FGF19 has to be assed during fasting in order to avid any change induced by meal consumption. In our systematic review, four studies (with a total of 897 patients) provided relevant information on the diagnostic accuracy of FGF19 with a positive cut-off ≤145 pg/mL. Compared to < 10% ^75^SeHCAT, the NPV and PPV of FGF19 the sensitivity and specificity of FGF19 as a marker of BAM ranged from 50 to 80% (mean 63.8%) and 65–79% (mean 72.3%), respectively. Pattni et al. [11] reported that NPV and PPV of FGF19 ≤ 145 pg/mL for a < 10% ^75^SeHCAT were 82 and 61%. Lenicek et al. found that FGF19 levels at a cut-off < 60 ng/L had a sensitivity and specificity of 80 and 68% in 466 patients with chronic diarrhea related to Crohn’s ileitis [22].

## Discussion

The present systematic review was conceived to collect the available data from the four major diagnostic tests, namely ^75^SeHCAT, C4, fecal BA assay and FGF19, proved to have clinical validity in identifying BAM in patients with chronic diarrhea due to various causes, mainly including functional (i.e., IBS-D, functional diarrhea) and inflammatory (Crohn’s disease) disorders. It should be noted, however, that data on BAM detection are still scarce and based mainly on small-size observational studies. Relevant calculation of the diagnostic accuracy of tests for BAM with true positive, true negative, false positive and false negative cases, which are essential parameters to assess sensitivity and specificity, have been reported in very few studies. Sensitivity and specificity were directly pooled from the included studies that reported diagnostic accuracy, and were shown as mean values in our systematic review. Nonetheless, because of the importance of this topic in daily practice and the possibility to improve the management of patients with chronic diarrhea, this systematic review aimed to demonstrate the actual diagnostic yield of few, though well established diagnostic tests for BAM, in patients with chronic diarrhea. Overall, data on ^75^SeHCAT, C4, fecal BA assay and FGF19 are encouraging although it should be stressed that any available diagnostic approach does not provide information as to whether BAs accumulate in the intestinal lumen as a result of true malabsorption or an increased secretion. Apart from this pathophysiological aspect, current available tests for BAM detection can provide objective information in patients with chronic diarrhea.

The final objective of studies involving tests for BAM should be the identification of high diagnostic accuracy, i.e. ‘biomarker(s)’, supporting the diagnosis of this underlying condition. The diagnostic test with the highest prevalence of positive results and highest diagnostic accuracy was ^75^SeHCAT retention, usually < 10% as a best cut-off. This is an external scintigraphic approach with two phases of detection, usually at 3 h and 7 days after oral administration of the 370 Kbq ^75^Se gamma-emitting synthetic BA (homocholic acid taurine). ^75^SeHCAT resulted to have an average sensitivity and specificity of 87.32% of 93.2% [21, 23, 24]. Our data confirm previous analysis. In fact, another systematic review with meta-analysis (36 studies with a total of 5028 patients with functional bowel disorder and diarrhea) estimating biomarkers for BAM, Valentin et al. [31] showed that < 10% ^75^SeHCAT (twenty-four studies) had the highest diagnostic yield (0.308 [0.247 to 0.377 CI]), followed by C4 (six studies) (0.171 [0.134 to 0.217 CI], fecal BAs at 48 h (two studies) (0.255 [0.0.071 to 0.606 CI] and, finally, FGF19 (three studies) (0.248 [0.147 to 0.385 CI].

^75^SeHCAT did not show good accuracy in detecting BAM in patients with ileal resection. In the study of Borghede et al. [32], the authors showed that ^75^SeHCAT yielded positive retention results indicative of BAM regardless the length of ileal resection. In fact, 39 out of 43 Crohn’s disease patients operated on ileal resection had a positive ^75^SeHCAT in a comparable percentage to that of Crohn’s disease but without ileal surgery. Hence, one can conclude that BAM-related chronic diarrhea in patients with Crohn’s ileitis occurs regardless ileal resection.

The most commonly used technique to determine BAM, ^75^SeHCAT, has some important limitations. First, this diagnostic approach shows several different thresholds of retention, which affect the results of the test. In a previous systematic review of 43 studies for a total of 1223 IBS patients, ^75^SeHCAT retention thresholds were as follows: 122 (10%) with < 5%; 339 (27%) with < 10% and 163 (13%) with < 15% retention [33]. In our evaluation of the diagnostic accuracy we selected a ^75^SeHCAT retention rate cut-off < 10%, which is most widely accepted for the diagnosis of BAM. Notably, a ^75^SeHCAT retention < 10% correlates with a faster colonic transit time [34]. This retention threshold is believed to perform best in order to identify BAM in patients with chronic diarrhea. Secondly, it is known that ^75^SeHCAT is not available in many countries apart from few tertiary referral centers. The limited availability of ^75^SeHCAT prompted research to other tests for BAM, which may be of more practical use. Thirdly, ^75^SeHCAT requires a nuclear medicine department, highly expensive equipment, trained personnel, it is time consuming for the patient (as the test consists of two phases of scintigraphic recording at day 0 and after 7 days) and, last but not least, it has an unavoidable radiation risk. Finally, about 50% of the published studies do not compare ^75^SeHCAT to the new diagnostic tests.

A previous systematic review addressed the cost-effectiveness analysis of the relationship between ^75^SeHCAT and response to cholestyramine treatment in patients with BAM. Only three studies, based on a limited number of patients, covered this topic and the results showed that, in the long-term, there were no consistent cost-effective differences among the main options including trial of treatment with cholestyramine; no use of ^75^SeHCAT; use of ^75^SeHCAT with a 15% cut-off [35]. Therefore, the ultimate choice is upon physicians and depending on resources available. Despite significant heterogeneity, in a recent meta-analysis, aimed to determine the proportion of patients with BAM amongst 361 cases with watery diarrhea and previous cholecystectomy, the authors showed a pooled BA diarrhea rate of 70% (95% CI 56–82%) regardless a 10% or 15% ^75^SeHCAT cut-off. Five out of eight studies (166 patients) demonstrated that cholestyramine treatment achieved a pooled response rate of 79% (95% CI 63 to 91%), thus confirming the usefulness of this BA sequestrant in patients with post-cholecystectomy associated BAM [36].

The C4 and FGF19 are the two serological tests, which can be assessed by HPLC and ELISA, respectively, to evaluate patients with BAM related chronic diarrhea. C4 yielded very positive results showing a mean reported sensitivity and specificity of 85.2 and 71.1%, respectively. Although with a lower diagnostic accuracy compared to ^75^SeHCAT, C4 assay is increasingly tested in patients with BAM because it is relatively simple, is not invasive, and exhibits an appreciable diagnostic accuracy [23].

Another serum marker of BAM is given by FGF19. The pathophysiological concept supporting this assay is based on the evidence that elevated levels of FGF19 inversely correlate with C4 expression, a mechanisms leading to inhibition of BA synthesis. Thus, low serum levels of FGF19 causes a reduced inhibition of BAs thereby promoting BAM and related diarrhea [22, 34, 37]. Lenicek et al. [22] demonstrated an indirect correlation between C4 (increased) and FGF19 (decreased) levels, suggesting that when combined these two techniques enhance the diagnostic sensitivity of BAM. The overall FGF19 sensitivity and specificity were 63.75 and 72.25%, with the ROC curve analysis showing that a cut-off of FGF19 < 145 pg/mL is a specific and sensitive marker for BAM [22, 34, 37]. FGF19 can be assessed by commercially available ELISA kits; it is an easy, non-invasive assay, and relatively not expensive diagnostic technique. However it should be emphasized that FGF19 levels show significant variations due to meal consumption. That is the reason why FGF19 should be assessed during fasting [16].

The evaluation fecal BA excretion is based on a high-fat (usually 100 g / day for 4 days) diet followed by the collection of the whole amount of feces in 48 h. HPLC is needed to evaluate BAs in fecal samples. Based on three studies, this test showed an overall sensitivity and specificity of 66.6 79.3%, thus lower compared to ^75^SeHCAT and C4, but higher than FGF19. Routine use of this diagnostic approach is limited by its laborious organization (i.e. patients should adhere to a high-fat diet, stools need to be collected in 48 h and, finally, BAs measured with HPLC, which is not readily available in any gastroenterological center) [14, 15]. Moreover, the amount of 100 g of dietary fat has been shown not to be able to evoke an increased entero-hepatic circulation in any patient / subject, thus undermining their actual effectiveness as challenge dose. Finally, some authors suggested a single fecal sample can be measured for BA assay to unravel patients with BAM, however this approach is still far from clinical application and further study is needed to test its diagnostic accuracy [14, 15, 31].

Our systematic review has two major drawbacks, which we would like to acknowledge. First, the lack of real gold standard tests for BAM represents the main reason hampering comprehensive meta-analysis studies comparing the diagnostic accuracy of available tests for BAM. Heterogeneity was high for all diagnostic tests, indicating that included patients with different etiologies of diarrhea caused a high degree of variability across the majority of studies, thus lowering the specificity of the BAM tests. Large specificity variation is also due to lack of gold standard tests. Secondly, very few studies (only 25%) compared head-to-head the available diagnostic methods for BAM diagnosis. In this line, it is important to point out that methods to assess patients with an underlying BAM are currently used in few tertiary referral centers. Despite these limitations, herein we provided clinically useful data about the average diagnostic accuracy reported from each included study and the expected prevalence of the positive test that can help physicians to the appropriate diagnostic work-up for patients with BAM related watery diarrhea.

## Conclusions

BAM is increasingly recognized as a possible cause contributing to diarrhea in patients with IBS-D, microscopic colitis, and even IBD with or without ileal inflammation. Consequently, there is a large unmet need in patients with chronic diarrhea for a better diagnosis and this is coupled with the need for better therapies. The test with the highest diagnostic accuracy for BAM is ^75^SeHCAT retention, although this test is not widely available in many countries, needs special equipment, has radiation exposure and it is expensive and time consuming for the patient. Serum C4 is a relatively simple blood test, accurate for patients who do not have liver disease or take statins. Also, C4 has a high diagnostic accuracy second only to ^75^SeHCAT. Fecal BA measurement provides an estimate of total BAs in the stool. Although data on this test come from one center, few studies have so far evaluated the diagnostic accuracy of this test. From the literature it seems to be a reasonable starting point to explore the use of FGF19 and C4 as relatively simple tests administrable to diagnose BAM in IBD patients. Patients with FGF19 values above > 145 ng/ml are unlikely to have BAM, and are unlikely to benefit from C4 testing, but those with values below this threshold should be investigated with C4 and/or ^75^SeHCAT where available. Secondary feedback down-regulation of colonic FXR expression represents a future option that needs validation in well designed, large cohort prospective studies enrolling patients with BAM. The novel tests for BAM related diarrhea, e.g. serum C4 and FGF19 as well as fecal BAs, should be more widely available and performed in any patients presenting with chronic diarrhea of unknown origin.

## Data Availability

Not applicable.

## Abbreviations

^75^SeHCAT: ^75^Selenium homotaurocholic acid test
AUROC: Area under receiver operating characteristic curve
BA: Bile acid
BAM: Bile acid malabsorption
C4: 7-α-hydroxy-4-cholesten-3-one
CYP7A1: Cholesterol-7-hydroxylase
FGF19: Fibroblast growth factor 19
FXR: Farsenoid X receptor
HPLC: High performance liquid chromatography
IBD: Inflammatory bowel disease
IBS-C: Constipation predominant irritable bowel syndrome
IBS-D: Diarrhea predominant irritable bowel syndrome
NPV: Negative predictive value
PPV: Positive predictive value
